# C8orf4 negatively regulates self-renewal of liver cancer stem cells via suppression of NOTCH2 signalling

**DOI:** 10.1038/ncomms8122

**Published:** 2015-05-19

**Authors:** Pingping Zhu, Yanying Wang, Ying Du, Lei He, Guanling Huang, Geng Zhang, Xinlong Yan, Zusen Fan

**Affiliations:** 1 School of Life Sciences, University of Science and Technology of China, Hefei, Anhui 230027, China; 2 Key Laboratory of Infection and Immunity of CAS, Institute of Biophysics, Chinese Academy of Sciences, 15 Datun Road, Chaoyang District, Beijing 100101, China; 3 Department of Hepatobiliary Surgery, PLA General Hospital, Beijing 100853, China; 4 University of Chinese Academy of Sciences, Beijing 100049, China

## Abstract

Liver cancer stem cells (CSCs) harbour self-renewal and differentiation properties, accounting for chemotherapy resistance and recurrence. However, the molecular mechanisms to sustain liver CSCs remain largely unknown. In this study, based on analysis of several hepatocellular carcinoma (HCC) transcriptome datasets and our experimental data, we find that C8orf4 is weakly expressed in HCC tumours and liver CSCs. C8orf4 attenuates the self-renewal capacity of liver CSCs and tumour propagation. We show that NOTCH2 is activated in liver CSCs. C8orf4 is located in the cytoplasm of HCC tumour cells and associates with the NOTCH2 intracellular domain, which impedes the nuclear translocation of N2ICD. C8orf4 deletion causes the nuclear translocation of N2ICD that triggers the NOTCH2 signalling, which sustains the stemness of liver CSCs. Finally, NOTCH2 activation levels are consistent with clinical severity and prognosis of HCC patients. Altogether, C8orf4 negatively regulates the self-renewal of liver CSCs via suppression of NOTCH2 signalling.

## Abstract (web summary)

Cancer stem cells are a small proportion of tumours that often confer resistance to chemotherapy but how these cells are maintained is unclear. Here, Zhu *et al*. study hepatocellular carcinoma stem cells and identify C8orf4 as a negative regulator of Notch2, which is required for the self-renewal of the cells.

## Graphical abstract

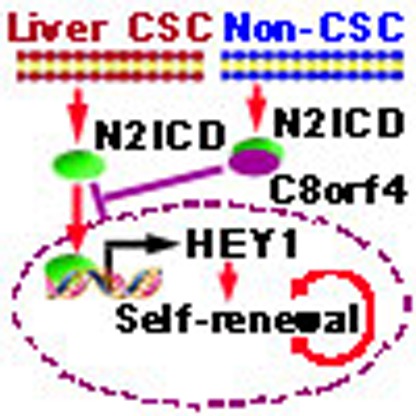

Hepatocellular carcinoma (HCC), the most common liver cancer, is the third leading cause of cancer related death^1^. The 5-year survival rate of HCC patients remains poor, and >750,000 HCC patients die each year. The high rate of recurrence and heterogeneity are the two major features of HCC^2^. Many studies have suggested that heterogeneity is a result of the hierarchical organization of tumour cells by a subset of cells with stem/progenitor cell features known as cancer stem cells (CSCs)^3^, which are cancer cells with stem cell features. These CSCs within tumour bulk display the capacity to self-renew, differentiate and give rise to a new tumour^4^, accounting for a hierarchical organization of heterogeneous cancer cells and a high rate of cancerous recurrence. Liver CSCs can be enriched by several defined surface markers, including epithelial cell adhesion molecule (EpCAM), CD13, CD133, CD90, CD24,CD44, calcium channel α2δ1 subunit and so on^5^,^6^,^7^,^8^.

Like stem cells, CSCs are characterized by self-renewal and differentiation simultaneously^9^. Not surprisingly, CSCs share core regulatory genes and developmental pathways with normal tissue stem cells. Accumulating evidence shows that NOTCH, Hedgehog and Wnt signalling pathways are implicated in the regulation of CSC self-renewal^4^. NOTCH signalling modulates many aspects of metazoan development and tissue stemness^10^,^11^. NOTCH receptors contain four members (NOTCH1–4) in mammals, which are activated by engagement with various ligands. The aberrant NOTCH signalling was first reported to be involved in the tumorigenesis of human T-cell leukaemia^12^,^13^. Recently, a number of studies have reported that the NOTCH signalling pathway is implicated in regulating self-renewal of breast stem cells and mammary CSCs^14^,^15^. However, how the NOTCH signalling regulates the liver CSC self-renewal remains largely unknown.

C8orf4, also called thyroid cancer 1 (TC1), was originally cloned from a papillary thyroid carcinoma and its surrounding normal thyroid tissue^16^. C8orf4 is ubiquitously expressed across a wide range of vertebrates with the sequence conservation across species. A number of studies have reported that C8orf4 is highly expressed in several tumours and implicated in tumorigenesis^17^,^18^,^19^. In addition, C8orf4 augments Wnt/β-catenin signalling in some cancer cells^20^,^21^, suggesting it may be involved in the regulation of self-renewal of CSCs. However, the biological function of C8orf4 in the modulation of liver CSC self-renewal is still unknown. Here we show that C8orf4 is weakly expressed in HCC and liver CSCs. NOTCH2 signalling is highly activated in HCC tumours and liver CSCs. C8orf4 negatively regulates the self-renewal of liver CSCs via suppression of NOTCH2 signalling.

## Results

### C8orf4 is weakly expressed in HCC tissues and liver CSCs

**Figure 1:**
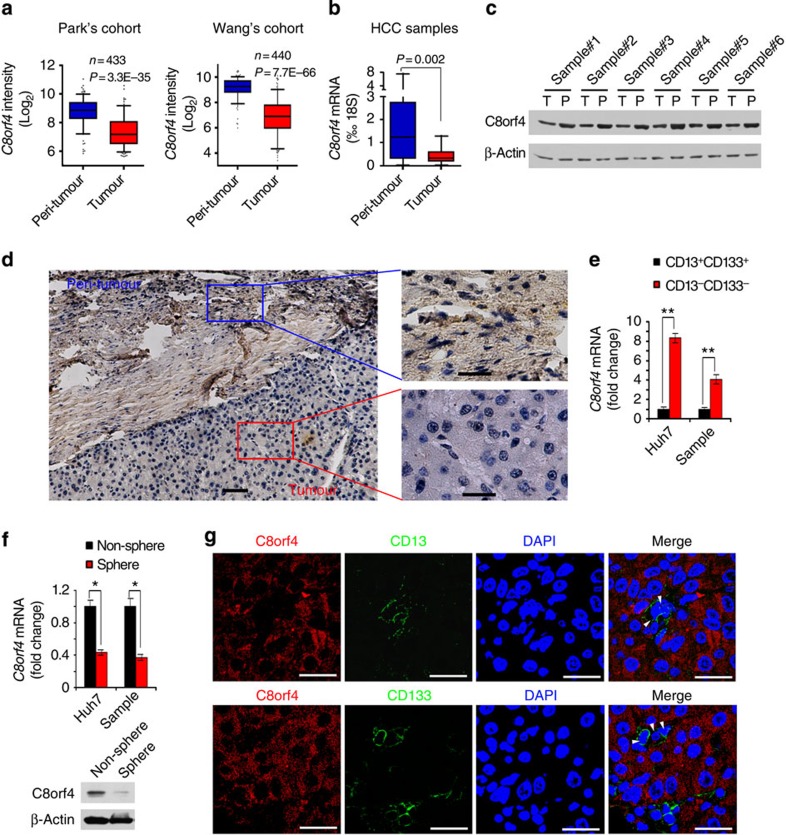
C8orf4 is weakly expressed in HCC tumours and liver CSCs. (**a**) *C8orf4* is weakly expressed in HCC patients. Using R language and Bioconductor methods, we analysed *C8orf4* expression in HCC tumour and peri-tumour tissues provided by Park's cohort (GSE36376) and Wang's cohort (GSE14520) datasets. (**b**,**c**) C8orf4 expression levels were verified in HCC patient samples by quantitative RT–PCR (qRT–PCR) (**b**) and immunoblotting (**c**). β-actin served as a loading control. 18S: 18S rRNA. (**d**) HCC samples were assayed by immunohistochemical staining. Scale bar—left: 50 μm; right: 20 μm. (**e**) *C8orf4* is weakly expressed in CD13^+^CD133^+^ cells sorted from Huh7 cells and primary HCC samples. *C8orf4* messenger RNA (mRNA) was measured by qRT–PCR. Six HCC samples got similar results. (**f**) *C8orf4* is much more weakly expressed in oncospheres than non-sphere tumour cells. Non-sphere: Huh7 or HCC primary cells that failed to form spheres. (**g**) HCC sample tissues were co-stained with anti-C8orf4 and anti-CD13 or anti-CD133 antibodies, then counterstained with DAPI for confocal microscopy. White arrows indicate CD13^+^ or CD133^+^ cells. Scale bars: 20 μm. For **a**,**b**, data are shown as box and whisker plot. Boxes represent interquartile range (IQR); upper and lower edge corresponds to the 75th and 25th percentiles, respectively. Horizontal lines within boxes represent median levels of gene intensity. Whiskers below and above boxes extend to the 5th and 95th percentiles, respectively. For **e** and **f**, Student's *t*-test was used for statistical analysis, *P<0.05; **P<0.01, data are shown as mean±s.d. Data are representative of at least three independent experiments. P, peri-tumour; T, tumour

To search for driver genes in the oncogenesis of HCC, we performed genome-wide analyses using several online-available HCC transcriptome datasets by R language and Bioconductor approaches. After analysing gene expression profiles of HCC tumour and peri-tumour tissues, we identified >360 differentially expressed genes from both Park's cohort (GSE36376; ref. 22) and Wang's cohort (GSE14520; refs 23, 24). Of these changed genes, we focused on *C8orf4*, which was weakly expressed in HCC tumours derived from both Park's cohort (GSE36376) and Wang's cohort (GSE14520) (Fig. 1a). Lower expression of C8orf4 was further confirmed in HCC samples by quantitative reverse transcription–PCR (qRT–PCR) and immunoblotting (Fig. 1b,c). In this study, HCC patient samples we used included all subtypes of HCC. In addition, these observations were further validated by immunohistochemical (IHC) staining (Fig. 1d). These data indicate that C8orf4 is weakly expressed in HCC tumour tissues.

Notably, *C8orf4* was also weakly expressed in embryonic stem cells (ESCs) and induced pluripotent stem cells (iPSCs) by analysis of its expression profiles derived from online datasets (GSE14897; ref. 25 and GSE25417; ref. 26) (Supplementary Fig. 1a,b). *C8orf4* was also lowly expressed in normal liver stem cells (Supplementary Fig. 1c,d), suggesting that C8orf4 may be involved in the regulation of self-renewal of liver stem cells. Thus, we propose that C8orf4 might play a role in the maintenance of liver CSCs. Since CD13 and CD133 were widely used as liver CSC surface markers, we sorted CD13^+^CD133^+^ cells from Huh7 and Hep3B HCC cell lines as well as HCC samples, serving as liver CSCs. We observed that *C8orf4* was weakly expressed in liver CSCs enriched from both HCC cell lines and patient samples (Fig. 1e). Six HCC samples were analysed for these experiments. Similar results were obtained in CD13^+^CD133^+^ cells from Hep3B cells. Furthermore, we performed sphere formation experiments using Huh7 cells and HCC primary sample cells, and detected expression levels of C8orf4. We observed that C8orf4 was dramatically reduced in the oncospheres generated by both HCC cell lines and patient samples (Fig. 1f). In addition, we noticed that C8orf4 expression was negatively correlated with liver CSC markers such as CD13 and CD133 in HCC samples (Fig. 1g), suggesting lower expression of C8orf4 in liver CSCs. Moreover, C8orf4 was mainly located in the cytoplasm of tumour cells. Altogether, C8orf4 is weakly expressed in HCC tumour tissues and liver CSCs.

### C8orf4 negatively regulates self-renewal of liver CSCs

**Figure 2:**
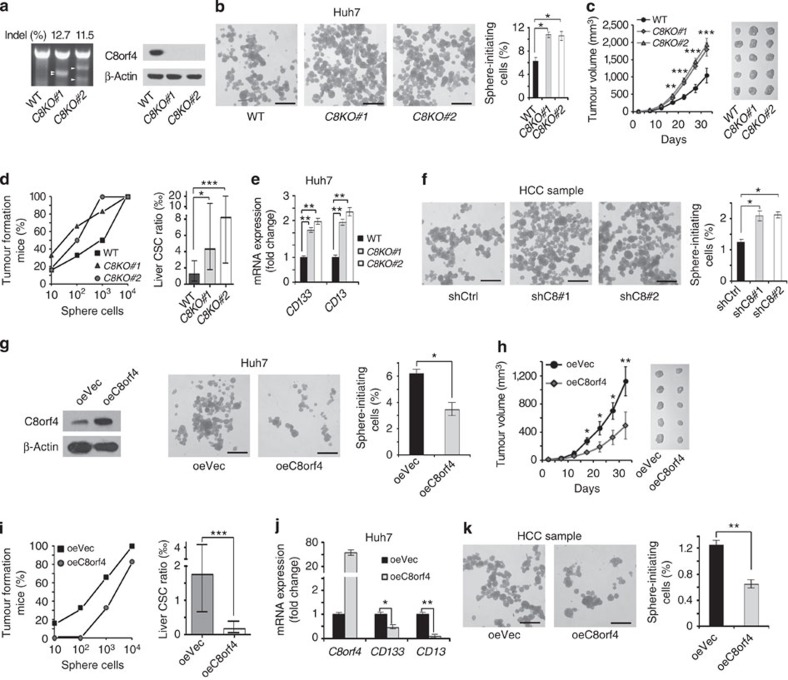
*C8orf4* knockout enhances self-renewal of liver CSCs. (**a**) *C8orf4*-deficient Huh7 cells were established using a CRISPR/Cas9 system. T7 endonuclease I cleavage confirmed the efficiency of sgRNA (left panel, white arrowheads), and *C8orf4*-knockout efficiency was confirmed by western blot (right panel). Two knockout cell lines were used. *C8KO#1*: *C8orf4KO#1*; *C8KO#2*: *C8orf4KO#2*. (**b**) *C8orf4*-deficient cells enhanced sphere formation activity. Calculated ratios are shown in the right panel. (**c**) *C8orf4*-deficient or WT Huh7 cells (1 × 10^6^) were injected into BALB/c nude mice. Tumour sizes were observed every 5 days. (**d**) *C8orf4* deficiency enhances tumour-initiating capacity. Diluted cell numbers of Huh7 cells were implanted into BALB/c nude mice for tumour initiation. Percentages of tumour-formation mice were calculated (left panel), and frequency of tumour-initiating cells was calculated using extreme limiting dilution analysis (right panel). Error bars represent the 95% confidence intervals of the estimation. (**e**) Expression levels of *CD13* and *CD133* were analysed in *C8orf4*-knockout Huh7 cells. (**f**) C8orf4 was silenced in HCC primary cells and C8orf4 depletion enhanced sphere formation activity. Calculated ratios are shown at the right panel. Three HCC specimens obtained similar results. (**g**) C8orf4-overexpressing Huh7 cells were established (left panel). C8orf4-overexpressing Huh7 cells and control Huh7 cells were cultured for sphere formation. (**h**,**i**) Xenograft tumour growth (**h**) and frequency of tumour-initiating cells (**i**) for C8orf4-overexpressing Huh7 cells were analysed as **c**,**d**. (**j**) C8orf4 overexpression reduces expression of *CD133* and *CD13* in Huh7 cells. (**k**) C8orf4 was transfected in HCC primary cells and cultured for sphere formation. Three HCC patient samples obtained similar results. Scale bars: **b**,**f**,**g**,**k**, 500 μm. Student's *t*-test was used for statistical analysis, *P<0.05; **P<0.01; ***P<0.001, data are shown as mean±s.d. Data represent at least three independent experiments. oeC8orf4, overexpression of C8orf4; oeVec, overexpression vector.

We then wanted to look at whether C8orf4 plays a critical role in the self-renewal maintenance of liver CSCs. *C8orf4* was knocked out in Huh7 cells through a CRISPR/Cas9 system (Fig. 2a). Two *C8orf4*-knockout (KO) cell strains were established and *C8orf4* was completely deleted in these two strains. *C8orf4* deletion dramatically enhanced oncosphere formation (Fig. 2b). We co-stained SOX9, a widely used progenitor marker, and Ki67, a well-known proliferation marker, in *C8orf4* KO sphere cells. We found that SOX9 was strongly stained in *C8orf4* KO sphere cells (Supplementary Fig. 2a). In contrast, Ki67 staining was not significantly altered in *C8orf4* KO sphere cells versus WT sphere cells. We also digested sphere cells and examined the SOX9 and Ki67 expression by flow cytometry. Similar results were achieved (Supplementary Fig. 2b). Importantly, through serial passage of CSC sphere cells, similar observations were obtained in the fourth generation oncosphere assay (Supplementary Fig. 2c,d). These data suggest that C8orf4 is involved in the regulation of liver CSC self-renewal.

In addition, *C8orf4*-deficient Huh7 cells overtly increased xenograft tumour growth (Fig. 2c). We then performed sphere formation and digested oncospheres formed by *C8orf4*-deficient or WT cells into single-cell suspension, then subcutaneously implanted 1 × 10^4^, 1 × 10^3^, 1 × 10^2^ and 10 cells into BALB/c nude mice. Tumour formation was examined for tumour-initiating capacity at the third month. *C8orf4* deficiency remarkably enhanced tumour-initiating capacity and liver CSC ratios (Fig. 2d). In addition, *C8orf4* deletion significantly enhanced expression levels of the liver CSC markers such as *CD13* and *CD133* (Fig. 2e). We also silenced C8orf4 in HCC primary cells using a lentivirus infection system and established C8orf4-silenced cells. Two pairs of short hairpin RNA (shRNA) sequences obtained similar knockdown efficiency. C8orf4 knockdown remarkably promoted sphere formation and xenograft tumour growth (Fig. 2f and Supplementary Fig. 2e). These data indicate that *C8orf4* deletion potentiates the self-renewal of liver CSCs.

We next overexpressed C8orf4 in Huh7 cells and HCC primary cells using lentivirus infection. We observed that C8orf4 overexpression in Huh7 cells remarkably reduced sphere formation and xenograft tumour growth (Fig. 2g,h). In addition, C8orf4 overexpression remarkably reduced tumour-initiating capacity and expression of liver CSC markers (Fig. 2i,j). Similar results were observed by C8orf4 overexpression in HCC primary cells (Fig. 2k). We tested three HCC samples with similar results. Overall, C8orf4 negatively regulates the maintenance of liver CSC self-renewal and tumour propagation.

### C8orf4 suppresses NOTCH2 signalling in liver CSCs

**Figure 3:**
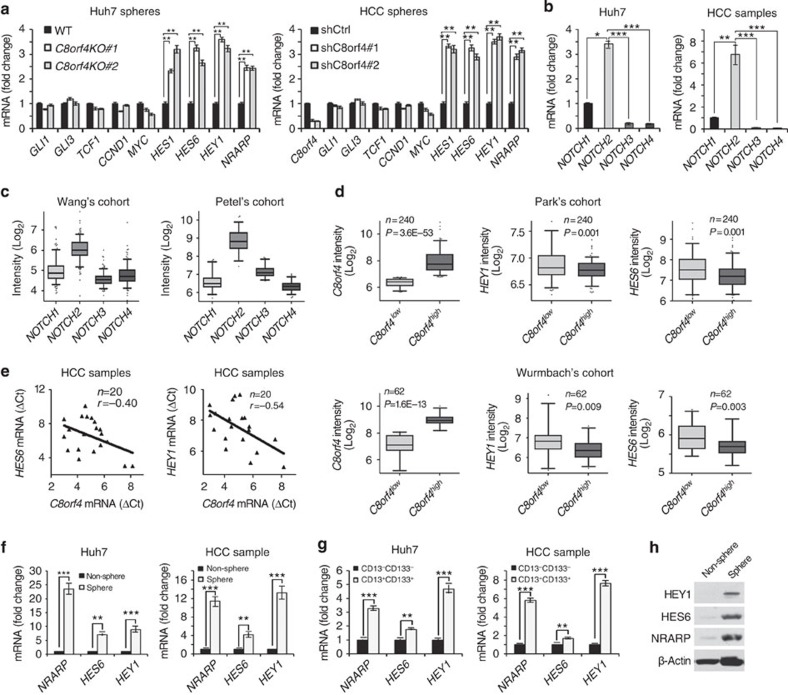
C8orf4 suppresses NOTCH2 signalling in liver CSCs. (**a**) *C8orf4* deficiency or depletion activates NOTCH signaling. The indicated major stemness signalling pathways were analysed in *C8orf4*-knockout Huh7 cells (left panel) and C8orf4-silenced primary cells of HCC samples (right panel). (**b**)Four receptor members of NOTCH family were examined in both Huh7 cells (left panel) and 29 pairs of HCC samples (right panel). (**c**) *NOTCH* receptors were analysed from Wang's cohort (left panel) and Petel's cohort (right panel) datasets. (**d**) *HEY1* and *HES6* were highly expressed in *C8orf4*^*low*^ samples by analysis of Park's cohort (upper panel) and Wurmbach's cohort (lower panel). (**e**) Expression levels of *HEY1* and *HES6* along with *C8orf4* were analysed in HCC samples by qRT–PCR. (**f**,**g**) Expression levels of *NRARP*, *HEY1* and *HES6* in spheres generated by Huh7 cells and HCC primary cells (**f**) and in CD13^+^CD133^+^ cells sorted from Huh7 cells and HCC primary cells (**g**). Non-sphere: Huh7 cells or HCC cells that failed to form spheres. (**h**) HEY1, HES6 and NRARP expression in sphere and non-sphere cells was detected by immunoblotting. β-actin was used as a loading control. For **c**,**d**, data are shown as box and whisker plot. Box: interquartile range (IQR); horizontal line within box: median; whiskers: 5–95 percentile. For **a**,**b**,**f**,**g**, Student's *t*-test was used for statistical analysis, *P<0.05; **P<0.01; ***P<0.001, data are shown as mean±s.d. Data are representative of at least three independent experiments.

To further determine the underlying mechanism of C8orf4 in the regulation of liver CSCs, we analysed three major self-renewal signalling pathways, including Wnt/β-catenin, Hedgehog and NOTCH pathways, in *C8orf4*-deleted Huh7 cells and HCC primary cells. We found that only NOTCH target genes were remarkably upregulated in *C8orf4*-deficient cells (Fig. 3a), whereas *C8orf4* deficiency did not significantly affect the Wnt/β-catenin or the Hedgehog pathway. Given that the NOTCH family receptors have four members, we wanted to determine which NOTCH member was involved in the C8orf4-mediated suppression of liver CSC stemness. We noticed that only *NOTCH2* was highly expressed in both Huh7 cells and HCC samples (Fig. 3b). In addition, this result was also confirmed by analysis of *NOTCH* expression levels derived from Wang's cohort (GSE14520) and Petel's cohort (E-TABM-36; ref. 27) (Fig. 3c). Moreover, we analysed expression profiles of *C8orf4* and *NOTCH* target genes using Park's cohort (GSE36376) and Wurmbach's cohort (GSE6764; ref. 28). These cohort datasets provided several Notch signalling and its target genes. *HEY1*, *NRARP* and *HES6* genes were highly expressed in HCC tumour tissues (GSE6764; ref. 28), which were further confirmed in HCC samples by real-time PCR (Supplementary Fig. 3a,b). Furthermore, *HEY1*, *NRARP* and *HES6* genes have been reported to be relatively specific NOTCH target genes. We then examined these three genes as the NOTCH2 target genes throughout this study. We found that the *C8orf4* expression level was negatively correlated with the expression levels of *HEY1* and *HES6*, suggesting that C8orf4 inhibited NOTCH signaling in HCC patients (Fig. 3d). Finally these results were further confirmed in HCC samples by qRT-PCR (Fig. 3e). To further explore the activation status of NOTCH2 signalling in liver CSCs, we examined the expression levels of NOTCH downstream target genes in oncospheres and CD13^+^CD133^+^ cells derived from both Huh7 cells and HCC cells. We observed that NOTCH target genes were highly expressed in liver CSCs (Fig. 3f,g). These observations were verified by immunoblotting (Fig. 3h). In addition, the expression levels of *NRARP*, *HES6* and *HEY1* were positively related to the expression levels of *EpCAM* and *CD133* derived from Zhang's cohort (GSE25097; ref. 29) and Wang's cohort (GSE14520; Supplementary Fig. 3c,d). These data suggest that the NOTCH2 signalling plays a critical role in the maintenance of self-renewal of liver CSCs.

### C8orf4 interacts with NOTCH2 that is critical for liver CSCs

**Figure 4:**
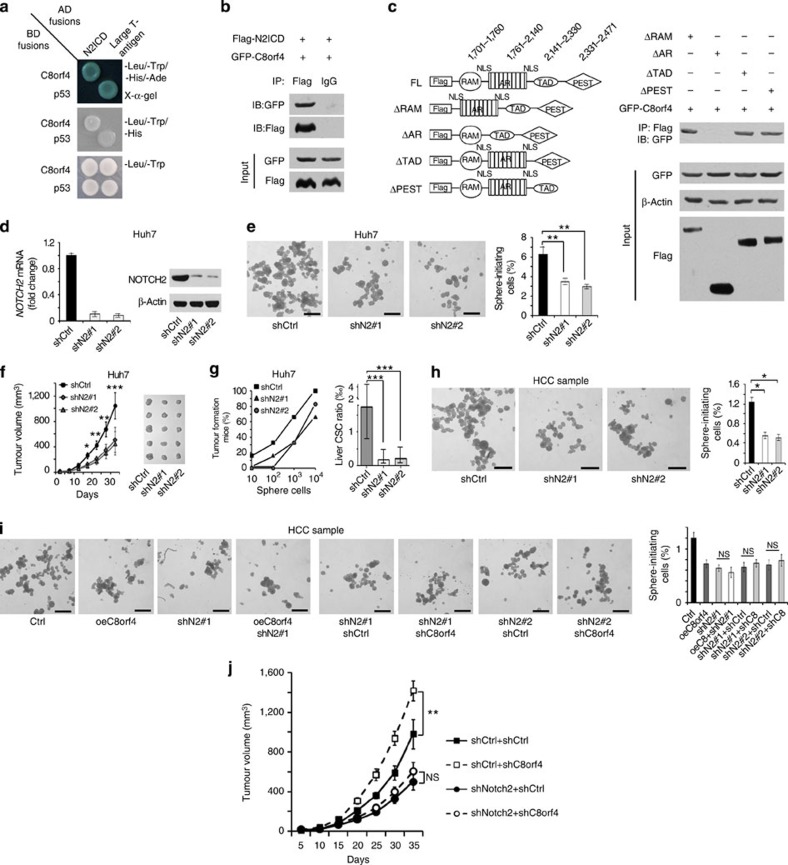
C8orf4 interacts with NOTCH2 that is required for the self-renewal maintenance of liver CSCs. (**a**) C8orf4 interacts with N2ICD. Yeast strain AH109 was co-transfected with Gal4 DNA-binding domain (BD) fused C8orf4 and Gal4-activating domain (AD) fused N2ICD. p53 and large T antigen were used as a positive control. (**b**) Recombinant Flag-N2ICD and GFP–C8orf4 were incubated for co-immunoprecipitation. (**c**) The ankyrin repeat AR domain is essential and sufficient for the interaction of C8orf4 with N2ICD. Various N2ICD truncation constructs were co-transfected with GFP–C8orf4 for domain mapping. NLS: nuclear location signal. (**d**) NOTCH2 was knocked down in Huh7 cells and detected by qRT–PCR and immunoblotting. (**e**) NOTCH2-silenced Huh7 cells were cultured for sphere formation assays. Two pairs of shRNAs against NOTCH2 obtained similar results. (**f**,**g**) Xenograft tumour growth (**f**) and frequency of tumour-initiating cells (**g**) for NOTCH2-silenced Huh7 cells were analysed. (**h**) NOTCH2 was silenced in HCC primary cells and NOTCH2 depletion declined sphere formation activity. Three HCC specimens obtained similar results. (**i**) Sphere formation capacity was examined in differently treated HCC primary cells. (**j**) HCC primary cells were treated with indicated lentivirus and implanted into BALB/c nude mice for xenograft tumour growth assays. Scale bars: **e**,**h**,**i**, 500 μm, Student's *t*-test was used for statistical analysis, *P<0.05; **P<0.01; ***P<0.001, data are shown as mean±s.d.. Data are representative of at least three independent experiments. IB, immunoblotting; IP, immunoprecipitation; NS, not significant.

On ligand–receptor binding, the NOTCH receptor experiences a proteolytic cleavage by metalloprotease and γ-secretase, releasing a NOTCH extracellular domain (NECD) and a NOTCH intracellular domain (NICD), respectively^30^. Then the active NICD undergoes nuclear translocation and activates the expression of NOTCH downstream target genes^31^.Then we constructed the NOTCH2 extracellular domain (N2ECD) and intracellular domain (N2ICD) and examined the interaction with C8orf4 via a yeast two-hybrid approach. Interestingly, we found that C8orf4 interacted with N2ICD, but not N2ECD (Fig. 4a). The interaction was validated by co-immunoprecipitation (Fig. 4b). Through domain mapping, the ankyrin repeat domain of NOTCH2 was essential and sufficient for its association with C8orf4 (Fig. 4c). Taken together, C8orf4 interacts with the N2ICD domain of NOTCH2.

To further verify the role of NOTCH2 in the maintenance of liver CSC self-renewal, we knocked down NOTCH2 in Huh7 cells and established stably depleted cell lines by two pairs of NOTCH2 shRNAs (Fig. 4d). NOTCH2 knockdown dramatically reduced sphere formation (Fig. 4e), as well as attenuated xenograft tumour growth and tumour-initiating capacity (Fig. 4f,g). Similar observations were achieved in NOTCH2-depleted HCC primary cells (Fig. 4h). In addition, we found that simultaneous knockdown of NOTCH2 and overexpression of C8orf4 failed to reduce sphere formation capacity compared with individual knockdown of NOTCH2 (Fig. 4i), suggesting that NOTCH2 and C8orf4 affected sphere formation through the same pathway. Meanwhile, C8orf4 knockdown failed to rescue the sphere formation ability of NOTCH2-depleted HCC primary cells (Fig. 4i). Similar observations were obtained in Huh7 cells (Supplementary Fig. 4). Finally, NOTCH2 depletion in C8orf4-silenced Huh7 cells or HCC primary cells also abrogated the C8orf4 depletion-mediated enhancement of xenograft tumour growth (Fig. 4j), suggesting that C8orf4 acted as upstream of NOTCH2 signalling. These data suggest that C8orf4 suppresses the liver CSC stemness through inhibiting the NOTCH2 signalling pathway.

### C8orf4 blocks nuclear translocation of N2ICD

**Figure 5:**
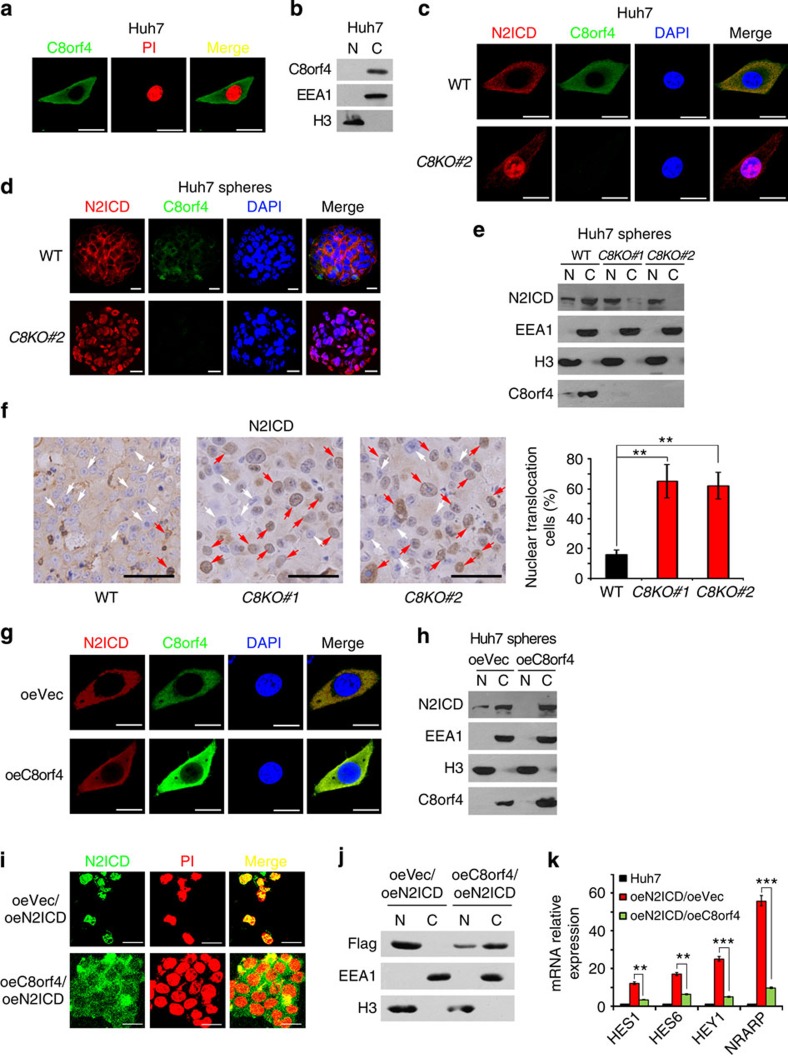
*C8orf4* deletion causes the nuclear translocation of N2ICD. (**a**) C8orf4 resides in the cytoplasm of Huh7 cells. Huh7 cells were permeabilized and stained with anti-C8orf4 antibody, then counterstained with PI for confocal microscopy. (**b**) Cellular fractionation was performed and detected by immunoblotting. (**c**,**d**) C8orf4 knockout causes the nuclear translocation of N2ICD. C8orf4-deficient Huh7 cells (**c**) and sphere cells (**d**) were permeabilized and stained with anti-C8orf4 and anti-N2ICD antibodies, then counterstained with DAPI followed by confocal microscopy. (**e**) Cellular fractionation was performed in C8orf4-deficient sphere and WT sphere cells followed by immunoblotting. (**f**) C8orf4-deficient Huh7 cells were implanted into BALB/c nude mice. Xenograft tumours were analysed by immunohistochemical staining. Red arrowheads denote nuclear translocation of N2ICD. (**g**) C8orf4-overexpressing Huh7 cells were permeabilized for immunofluorescence staining. (**h**) Cellular fractionation was performed in C8orf4-overexpressing Huh7 cells for immunoblotting. (**i**,**j**) C8orf4 was overexpressed in N2ICD-overexpressing Huh7 cells followed by immunofluorescence staining (**i**) and immunoblotting (**j**). (**k**) NOTCH target genes were measured in cells treated as in **i** by real-time PCR. Scale bars: **a**,**c**,**d**,**g,i**, 10 μm; **f**, 40 μm. Student's *t*-test was used for statistical analysis, **P<0.01; ***P<0.001, data are shown as mean±s.d.. Data represent at least three independent experiments.

As shown in Fig. 1g, C8orf4 was mainly localized in the cytoplasm in tumour cells of HCC samples. To confirm these observations, we stained C8orf4 in several HCC cell lines and noticed that C8orf4 also resided in the cytoplasm of Huh7 cells and Hep3B cells (Fig. 5a and Supplementary Fig. 5a). These results were further validated by cellular fractionation (Fig. 5b). Importantly, *C8orf4* KO led to nuclear translocation of N2ICD (Fig. 5c). In addition, we also examined the intracellular location of N2ICD in Huh7 spheres. We found that *C8orf4* deletion caused complete nuclear translocation of N2ICD in oncosphere cells (Fig. 5d,e), while N2ICD was mainly located in the cytoplasm of WT oncosphere cells. However, we found that *C8orf4* KO did not affect subcellular localization of β-catenin (Supplementary Fig. 5b,c). Through luciferase assays, C8orf4 transfection did not significantly influence promoter transcription activity of Wnt target genes such as *TCF1*, *LEF* and *SOX4* (Supplementary Fig. 5d). These data indicate that C8orf4 resides in the cytoplasm of HCC cells and inhibits nuclear translocation of N2ICD.

To further determine whether C8orf4 inhibits the NOTCH2 signalling in the propagation of xenograft tumours, we examined the distribution of N2ICD and NOTCH2 target gene activation in *C8orf4*-deficient xenograft tumour tissues. We found that *C8orf4*-deficient tumours displayed much more nuclear translocation of N2ICD compared with WT tumours (Fig. 5f). Expectedly, *C8orf4*-deficient tumours showed elevated expression levels of NOTCH2 target genes such as *HEY1*, *HES6* and *NRARP* (Supplementary Fig. 5e). Furthermore, C8orf4 overexpression blocked the nuclear translocation of N2ICD (Fig. 5g,h). Consequently, C8orf4-overexpressing tumours showed much less N2ICD nuclear translocation and reduced expression levels of NOTCH2 target genes compared with control tumours (Supplementary Fig. 5f,g). Of note, C8orf4 overexpression in N2ICD-overexpressing Huh7 cells still blocked nuclear translocation of N2ICD (Fig. 5i,j). Consequently, C8orf4 overexpression abolished the activation of Notch2 signalling (Fig. 5k). These results suggest that *C8orf4* deletion causes the nuclear translocation of N2ICD leading to activation of NOTCH2 signalling.

### NOTCH2 signalling is required for the stemness of liver CSCs

**Figure 6:**
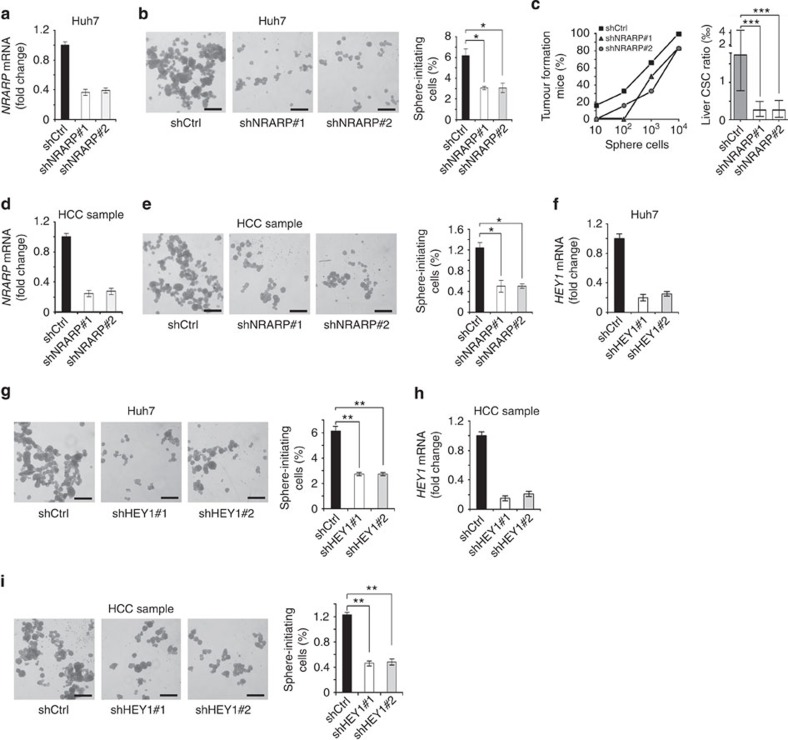
Depletion of NRARP and HEY1 impairs stemness of liver CSCs. (**a**,**b**) NRARP-silenced Huh7 cells were established (**a**) and showed reduced sphere formation capacity (**b**). Two pairs of shRNAs against NRARP obtained similar results. (**c**) NRARP-silenced Huh7 cells decline tumour-initiating capacity (left panel) and reduce liver CSC frequency (right panel). Error bars represent the 95% confidence intervals of the estimation. (**d**,**e**) NRARP was knocked down in HCC primary cells (**d**) and sphere formation was detected (**e**). Three HCC samples were tested with similar results. (**f**,**g**) HEY1-silenced Huh7 cells were established (**f**) and sphere formation was assayed (**g**). Two pairs of shRNAs against HEY1 obtained similar results. (**h**,**i**) HEY1 was knocked down in HCC primary cells (**h**) and HEY1 depletion impaired sphere formation capacity (**i**). Three HCC samples were tested with similar results. Scale bars: **b**,**e**,**g**,**i**, 500 μm. For **a**,**b**,**d**–**i**, Student's *t*-test was used for statistical analysis, *P<0.05; **P<0.01; ***P<0.001, data are shown as mean±s.d. Data are representative of at least three independent experiments.

To further verify the role of NRARP and HEY1 in the maintenance of liver CSC self-renewal, we knocked down these two genes in Huh7 cells and established stably depleted cell lines by two pairs of shRNAs. As expected, NRARP knockdown dramatically reduced sphere formation (Fig. 6a,b). NRARP knockdown also attenuated tumour-initiating capacity and liver CSC ratios (Fig. 6c). Similar results were achieved in NRARP-silenced HCC primary cells (Fig. 6d,e). Similarly, HEY1 silencing remarkably reduced sphere formation derived from Huh7 and HCC primary cells (Fig. 6f–i), as well as declined xenograft tumour growth and tumour-initiating capacity (Supplementary Fig. 6a,b). In sum, NOTCH2 signalling is required for the maintenance of liver CSC self-renewal.

### NOTCH2 signalling is correlated with HCC severity

**Figure 7:**
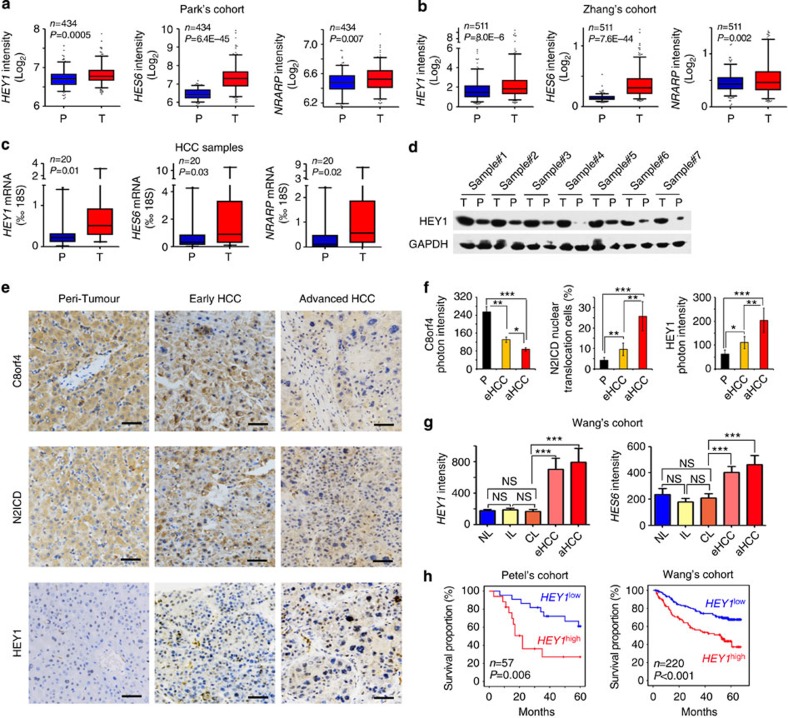
NOTCH2 activation levels are consistent with clinical severity and prognosis of HCC patients. (**a**,**b**) NOTCH target genes were highly expressed in HCC tumour tissues derived from Park's cohort (**a**) and Zhang's cohort (**b**). (**c**) High expression levels of NOTCH target genes in HCC tumour tissues were verified by qRT–PCR. (**d**) HEY1 expression in HCC tumour tissues was detected by western blot. (**e**) IHC staining for N2ICD, C8orf4 and HEY1. These images represent 93 HCC samples. Scale bars, 50 μm. (**f**) IHC images were calculated using Image-Pro Plus 6. (**g**) Expression levels of NOTCH target genes were elevated in HCC tumours and advanced HCC patients derived from Wang's cohort. (**h**) *HEY1* expression level was positively correlated with prognosis prediction of HCC patients analysed by Petel's cohort and Wang's cohort. HCC samples were divided into two groups according to *HEY1* expression levels followed by Kaplan–Meier survival analysis. For **a**–**c**, data are shown as box and whisker plot, Box: interquartile range (IQR); horizontal line within box: median; whiskers: 5–95 percentile. For **f**,**g**, Student's *t*-test was used for statistical analysis, *P<0.05; **P<0.01; ***P<0.001; data are shown as mean±s.d. Experiments were repeated at least three times. aHCC, advanced HCC; CL, cirrhosis liver; eHCC, early HCC; IL, inflammatory liver; NL, normal liver; NS, not significant.

As shown above, the NOTCH2 signalling was highly activated in liver CSCs and involved in the regulation of liver CSC stemness. We further examined the relationship of NOTCH2 signalling with the progression of HCC. First, we analysed NOTCH2 activation levels in HCC tumour tissues and peri-tumour tissues derived from Park's cohort (GSE36376). We observed that *HEY1*, *HES6* and *NRARP* were highly expressed in the tumour tissues of HCC patients (Fig. 7a). Consistently, high expression levels of *HEY1*, *HES6* and *NRARP* in HCC tumours were validated by Zhang's cohort (GSE25097) (Fig. 7b). Importantly, high expression of these three genes was confirmed in HCC samples through quantitative RT–PCR (Fig. 7c), as well as immunoblotting (Fig. 7d). To confirm a causative link between low C8orf4 expression level and nuclear N2ICD, we examined 93 HCC samples (31 peri-tumour, 37 early stage of HCC patients and 25 advanced stage of HCC patients) with immunohistochemistry staining. We observed that nuclear staining of N2ICD appeared in ∼10% tumour cells in the majority of early HCC patients we tested (Fig. 7e,f). In advanced HCC patients, nuclear staining of N2ICD in tumour cells increased to ∼30% in almost all the advanced HCC patients we examined. Consequently, HEY1 staining existed in ∼10% tumour cells with scattered distribution and increased to 30% tumour cells in the advanced HCC patients (Fig. 7e). Consistently, low expression of C8orf4 was well correlated with activation of NOTCH2 signalling (Fig. 7e,f).

Serial passages of colonies or sphere formation *in vitro*, as well as transplantation of tumour cells, are frequently used to assess the long-term self-renewal capacities of CSCs^32^. We used HCC primary cells for serial passage growth *in vitro* and tested the expression levels of *C8orf4*, *HEY1* and *SOX9*. We found that *C8orf4* expression was gradually reduced over serial passages in oncosphere cells (Supplementary Fig. 7a). Consequently, the expression of NOTCH2 targets such as *HEY1* and *SOX9* was gradually increased in oncosphere cells during serial passages (Supplementary Fig. 7b). In addition, N2ICD nuclear translocation appeared in oncosphere cells with high expression of HEY1 plus low expression of C8orf4 (termed as C8orf4^−^/N2ICDnuc/HEY1^+^ cells) (Supplementary Fig. 7c). These data suggest that the C8orf4^−^/N2ICDnuc/HEY1^+^ fraction cells represent a subset of liver CSCs.

Through analysing Wang's cohort (GSE54238), we noticed that the NOTCH2 activation levels were positively correlated with the development and progression of HCC (Fig. 7g). By contrast, the NOTCH2 pathway was not activated in inflammation liver, cirrhosis liver and normal liver (Fig. 7f). Consistently, similar observations were achieved by analysis of Zhang's cohort (GSE25097) (Supplementary Fig. 7d). In addition, the NOTCH2 activation levels were consistent with clinicopathological stages of HCC patients derived from Wang's cohort (GSE14520) (Supplementary Fig. 7e). Finally, HCC patients with higher expression of *HEY1* displayed worse prognosis derived from Petel's cohort (E-TABM-36) and Wang's cohort (GSE14520) (Fig. 7h). These two cohorts (E-TABM-36 and GSE14520) have survival information of HCC patients. Taken together, the NOTCH2 activation levels in tumour tissues are consistent with clinical severity and prognosis of HCC patients.

## Discussion

CSC have been identified in many solid tumours, including breast, lung, brain, liver, colon, prostate and bladder cancers^4^,^6^,^33^. CSCs have similar characteristics associated with normal tissue stem cells, including self-renewal, differentiation and the ability to form a new tumour. CSCs may be responsible for cancer relapse and metastasis due to their invasive and drug-resistant capacities^34^. Thus, targeting CSCs may become a promising therapeutic strategy to deadly malignancies^35^,^36^. However, it remains largely unknown about hepatic CSC biology. In this study, we used CD13 and CD133 to enrich CD13^+^CD133^+^ subpopulation cells as liver CSCs. Based on analysis of several online-available HCC transcriptome datasets, we found that C8orf4 is weakly expressed in HCC tumours as well as in CD13^+^CD133^+^ liver CSCs. NOTCH2 signalling is required for the maintenance of liver CSC self-renewal. C8orf4 resides in the cytoplasm of tumour cells and interacts with N2ICD, blocking the nuclear translocation of N2ICD. Lower expression of C8orf4 causes nuclear translocation of N2ICD that activates NOTCH2 signalling in liver CSCs. NOTCH2 activation levels are consistent with clinical severity and prognosis of HCC patients. Therefore, C8orf4 negatively regulates self-renewal of liver CSCs via suppression of NOTCH2 signalling.

Elucidating signalling pathways that maintains self-renewal of liver CSCs is pivotal for the understanding of hepatic CSC biology and the development of novel therapies against HCC. Several signalling pathways, such as Wnt/β-catenin, transforming growth factor-beta, AKT and STAT3 pathways, have been defined to be implicated in the regulation of liver CSCs^37^. Not surprisingly, some liver CSC subsets and normal tissue stem cells may share core regulatory genes and common signalling pathways. The NOTCH signalling pathway plays an important role in development via cell-fate determination, proliferation and cell survival^38^,^39^. The NOTCH family receptors contain four members in mammals (NOTCH1–4), which are activated by binding to their corresponding ligands. A large body of evidence provides that NOTCH signalling is implicated in carcinogenesis^40^. However, the role of NOTCH signalling in liver cancer is controversial. A previous study reported that NOTCH1 signalling suppresses tumour growth of HCC^41^. Recently, several reports showed that NOTCH signalling enhances liver tumour initiation^42^,^43^,^44^. Importantly, a recent study showed that various NOTCH receptors have differential functions in the development of liver cancer^45^. Here we demonstrate that NOTCH2 signalling is activated in HCC tumour tissues and liver CSCs, which is required for the maintenance of liver CSC self-renewal.

C8orf4, also known as TC1, was originally cloned from a papillary thyroid cancer^16^,^46^. The copy number variations of C8orf4 are associated with acute myeloid leukaemia and other haematological malignancies^19^,^47^. C8orf4 has been reported to be implicated in various cancers. C8orf4 was highly expressed in thyroid cancer, gastric cancer and breast cancer^16^,^20^,^46^. C8orf4 has been reported to enhance Wnt/β-catenin signalling in cancer cells that is associated with poor prognosis^20^,^21^. However, C8orf4 is downregulated in colon cancer^48^. In this study, we show that C8orf4 is weakly expressed in HCC tumour tissues and liver CSCs. Our observations were confirmed by two HCC cohort datasets. Importantly, C8orf4 negatively regulates the NOTCH2 signalling to suppress the self-renewal of liver CSCs. Therefore, C8orf4 may exert distinct functions in the regulation of various malignancies.

NOTCH receptors consist of noncovalently bound extracellular and transmembrane domains. Once binding with membrane-bound Delta or Jagged ligands, the NOTCH receptors undergoes a proteolytic step by metalloprotease and γ-secretase, generating NECD and NICD fragments^11^,^31^. The NICD, a soluble fragment, is released in the cytoplasm on proteolysis. Then the NICD translocates to the nucleus and binds to the transcription initiation complex, leading to activation of NOTCH-associated target genes^49^. However, it is largely unclear how the NICD is regulated during NOTCH signalling activation. Here we show that N2ICD binds to C8orf4 in the cytoplasm of liver non-CSC tumour cells, which impedes the nuclear translocation of N2ICD. By contrast, in liver CSCs, lower expression of C8orf4 causes the nuclear translocation of N2ICD, leading to activation of NOTCH signalling.

CSCs or tumour-initiating cells, behave like tissue stem cells in that they are capable of self-renewal and of giving rise to hierarchical organization of heterogeneous cancer cells^4^. Thus, CSCs harbour the stem cell properties of self-renewal and differentiation. Actually, the CSC model cannot account for tumorigenesis in all tumours. CSCs could undergo genetic evolution, and the non-CSCs might switch to CSC-like cells^4^. These results highlight the dynamic nature of CSCs, suggesting that the clonal evolution and CSC models can act in concert for tumorigenesis. Furthermore, low C8orf4 expression in tumour cells results in overall Notch2 activation, which then may have more of a progenitor signature and be more aggressive. These cells would likely have a growth advantage in non-adherent conditions and express many of the stemness markers. The dynamic nature of CSCs or persistent NOTCH2 activation may contribute to the high number of C8orf4^−^/N2ICDnuc/HEY1^+^ cells in advanced HCC tumours and correlation in the patient cohort.

A recent study showed that NOTCH2 and its ligand Jag1 are highly expressed in human HCC tumours, suggesting activation of NOTCH2 signalling in HCC^45^. In addition, inhibiting NOTCH2 or Jag1 dramatically reduces tumour burden and growth. However, suppression of NOTCH3 has no effect on tumour growth. Dill *et al*.^43^ reported that Notch2 is an oncogene in HCC. Notch2-driven HCC are poorly differentiated with a high expression level of the progenitor marker Sox9, indicating a critical role of Notch2 signalling in liver CSCs. Here we found that NOTCH2 and its target genes such as NRARP, HEY1 and HES6 are highly expressed in HCC samples. In addition, depletion of NRARP and HEY1 impairs the stemness maintenance of liver CSCs and tumour propagation. Moreover, the expression levels of NRARP, HEY1 and HES6 in tumours are positively correlated with clinical severity and prognosis of HCC patients. Finally, the NOTCH2 activation status is positively related to the clinicopathological stages of HCC patients. Altogether, C8orf4 and NOTCH2 signalling can be detected for the diagnosis and prognosis prediction of HCC patients, as well as used as targets for eradicating liver CSCs for future therapy.

## Methods

### Cell lines and tumour specimens

Human liver cancer cell lines Huh7 and Hep3B were provided by Dr Zeguang Han (the Shanghai Jiaotong University School of Medicine, Shanghai, China). Cells were maintained in DMEM supplemented with 10% FBS (Life Technologies), 100 μg ml^−1^ penicillin and 100 U ml^−1^ streptomycin. Human liver cancer specimens were obtained from the Department of Hepatobiliary Surgery, PLA General Hospital (Beijing, China) with informed consent, according to the Institutional Review Board approval. We numbered HCC samples according to obtaining date, and randomly utilized the samples without artificial bias.

### Antibodies and reagents

Commercial antibodies were mouse anti-β-actin, mouse anti-Flag, rabbit anti-C8orf4 antibodies (Sigma-Aldrich); mouse anti-green fluorescent protein (anti-GFP), PE-conjugated CD13 antibodies (Sungene Biotech, Tianjin); rabbit anti-NOTCH2 antibody (Cell Signaling Technology); PE-conjugated CD133 antibody (BD), Rabbit anti-CD133, anti-CD13 antibodies (Sangon Biotech, Shanghai); Calreticulin, Alexa488-conjugated goat anti-rat immunoglobulin-G (IgG; Biolegend); Alexa488-conjugated donkey anti-mouse IgG, Alexa594-conjugated donkey anti-mouse IgG, Alexa594-conjugated donkey anti-rabbit IgG (Molecular Probes) and horseradish peroxidase (HRP)-conjugated secondary antibody (Santa Cruz). Dilutions of anti-β-actin, anti-Flag, anti-GFP and HRP-conjugated secondary antibodies (for western blot) were 1:5,000, dilutions of anti-CD133, anti-CD13 and fluorescence-conjugated or HRP-conjugated secondary antibodies (for IHC staining) were 1:500, and the others were 1:1,000. Other major reagents were bFGF (Millipore), EGF (Sigma-Aldrich), N2 supplement (Life Technologies), B27 (Life Technologies), PEG8000 (Sigma-Aldrich), DAPI (4′,6-diamidino-2-phenylindole; Sigma-Aldrich) and PI (Sigma-Aldrich).

### Quantitative RT–PCR

Extraction of specimen RNA was followed by a standard protocol provided by Life Technology. Briefly, fresh specimens were homogenized with Trizol, and then segregated by adding chloroform, after obtaining the aqueous phase isopropanol and 75% ethanol were added sequentially. RNA pellet was resolved in RNase free H_2_O. After RT of messenger RNA by a standard protocol provided by Promega, complementary DNA was obtained and used as a template for quantitative PCR. Quantitative PCR kit was purchased from TIANGEN BioMart (Beijing) and sequence-specific primers were obtained from SanGon Company (Shanghai). We examined messenger RNA expression by quantitative PCR with ABI7300 (Applied Biosystems). Sequences of PCR primers are shown in the Supplementary Table 1.

### Western blot

For western blot, tumour cells or specimens were homogenized with RIPA buffer (150 mM NaCl, 0.5% sodium deoxycholate, 0.1% SDS, 1% NP40, 1 mM EDTA, 50 mM Tris (pH 8.0)). After boiling for 15 min, supernatants were loaded onto SDS–PAGE gels. Nitrocellulose membranes were incubated with primary antibodies for immunoblotting, then incubated and visualized by HRP-conjugated secondary antibodies. We provided uncropped scans of western blot in Supplementary Fig. 8.

### Co-immunoprecipitation

For co-immunoprecipitation, oncospheres were harvested, digested with trypsin/EDTA and washed three times with PBS, then treated with RIPA buffer for 30 min at 4 °C. Precipitation was removed from cell lysates by centrifugation at 14,000*g* for 10 min and the supernatant was precleared by protein A/G beads (Santa Cruz Biotechnology). After 1 h incubation, precleared protein A/G beads were removed and primary antibody was added for overnight incubation, and new protein A/G beads were added for 3 h incubation, then beads were collected and washed with PBS. Finally samples were subjected onto SDS–PAGE gels and detected by western blot.

### Flow cytometry

For flow cytometry, cells were digested with trypsin/EDTA, after being labelled with proper primary antibodies and secondary antibodies samples were washed three times, then loaded and analysed with FACSAriaIl. Compared with IgG control sample, antibody-specific staining cells and negative cells were gated and collected by adding magnetic field. Then sorted cells were digested for qRT–PCR to detect gene expression levels.

### CRISPR/Cas9 KO system

C8orf4-deficient Huh7 cells were established using CRISPR/Cas9 according to standard protocol provided by Zhang's lab^50^. Briefly, sgRNA was generated by online CRISPR Design Tool (http://tools.genome-engineering.org) and cloned into pSpCas9(BB)-2A–GFP vector. After confirming cutting efficiency of sgRNA, pSpCas9 vectors were transfected into Huh7 cells. Sorted GFP-positive Huh7 cells were seeded into 96-well plates for monoclonalization. Four weeks later, the cell clones derived from single cells were detected for gene expression. For C8orf4 KO, two pairs of small guide RNA (sgRNA) were used, and their sequences were 5′- TGGGCTGACTCGTAGCGACG -3′ and 5′- GCCCACGGCTTTCTTACGAG -3′.

### Yeast two-hybrid assay

Yeast two-hybrid assay was performed using Matchmaker Gold Yeast Two-Hybrid system (Takara Bio Inc.). Briefly, C8orf4 was subcloned into pGBKT7 plasmid (BD-C8orf4). N2ICD and N2ECD were subcloned into pGADT7 plasmid (AD-N2ICD and AD-N2ECD). BD-C8orf4 and AD-N2ICD or AD-N2ECD were co-transformed into AH109 cells, and then plated on SD plate lacking adenine, histidine, tryptophan and leucine^51^. Selected clones were detected for β-galactosidase activity.

### IHC staining

Formalin-fixed HCC sections were deparaffinized using xylene and then rehydrated with graded alcohols and finally distilled water. After being treated with 3% H_2_O_2_ for 15 min, the slides were treated for antigen retrieval in 121 °C for 5 min, and then cooled down to room temperature slowly. After 30 min incubation in 10% goat serum, the sections were incubated in proper primary antibodies (C8orf4, N2ICD and HEY1, 1:1,000 dilution) overnight. After washing three times with PBS, the sections were incubated in HRP-conjugated secondary antibodies (1:500 dilution), and the subsequent detection was performed using the standard substrate detection of HRP. Then the sections were stained with haematoxylin and dehydration in graded alcohols and xylene. For C8orf4 staining, citrate/sodium citrate buffer was used for antigen retrieval. For HEY1 and N2ICD staining, Tris-EDTA buffer (10 mM, pH 8.0) was used for antigen retrieval.

### Lentivirus production and cell infection

We constructed pBPLV–GFP vector and Psin–GFP–C8orf4 plasmid, and transfected 293T cells for virus production. Huh7 and HCC primary cells were infected by the virus supernatants. After sorting for GFP-positive cells, we established C8orf4 overexpression cells. C8orf4, NOTCH2-, NRARP- and HEY1-silenced Huh7 or HCC primary cells were established using pSicoR–GFP shRNA vectors by a similar strategy. For HCC sample cells, we used high infection efficiency lentivirus containing pBPLV–GFP overexpression vector or pSicoR shRNA vector. shRNA sequences are listed in the Supplementary Table2.

### Sphere formation assay

Thousand Hep3B cells or 5,000 HCC primary cells were seeded in Ultra Low Attachment 6-well plates (Corning Incorporated Life Sciences, Acton, MA, USA) and cultured in Dulbecco's modified Eagle's medium/F12 (Life Technologies) supplemented with B27, N2, 20 ng ml^−1^ epidermal growth factor and 20 ng ml^−1^ basic fibroblast growth factor (Millipore). Cells were incubated in a CO_2_ incubator, and 2 weeks later spheres were counted under stereomicroscope (Olympus, Tokyo, Japan). The spheres were fixed for immunofluorescence staining or digested for co-immunoprecipitation and western blot assays. For non-sphere cell separation, we collected sphere formation medium that contains non-sphere cells and sphere cells in an Eppendorf tube and let stand for 5 min and pellets were spheres. Supernatants were then removed in a new Eppendorf tube carefully with transferpettor and collected by centrifugation at 1,500*g* for 5 min. Pellets were non-sphere cells and used directly for subsequent experiments. We cultured these non-sphere cells under the same non-adherent conditions as sphere cells.

### *In vivo* xenograft experiments

For tumour growth assays, 1 × 10^6^ tumour cells were injected into 6-week-old male BALB/c nude mice. Every 5 days, tumour volume was calculated by the formula *V*=*πab*^2^/6 (*a*: tumour length, *b*: tumour width). For tumour-formation assay, spheres were digested in single cells by trypsin/EDTA, and 10, 10^2^, 10^3^ and 10^4^ cells were injected into 6-week-old male BALB/c nude mice. Tumour formation was observed every month and analysed at the third months. BALB/c nude mice were obtained from the Animal Centre of the Chinese Academy of Medical Sciences (Beijing, China), and all experiments involving mice were approved by the institutional committee of Institute of Biophysics, Chinese Academy of Sciences.

### Statistical analysis

Cohort datasets were downloaded from NCBI or EBI. R language and Bioconductor methods were used for background correction, normalization, calculation of gene expression and annotation^52^. Gene and expression lists generated by R3.1.0 were used for further analysis. Tumour-initiating cell frequency was calculated using extreme limiting dilution analysis^53^.

## Additional information

**How to cite this article:** Zhu, P. *et al*. C8orf4 negatively regulates self-renewal of liver cancer stem cells via suppression of NOTCH2 signalling. *Nat. Commun.* 6:7122 doi: 10.1038/ncomms8122 (2015).

## Supplementary Material

Supplementary Information Supplementary Figures 1-8, Supplementary Tables 1-2
