# Supplementary material for: C8orf4 negatively regulates self-renewal of liver cancer stem cells via suppression of NOTCH2 signalling
Source: Nat Commun. 2015 May 19;6:7122. doi: 10.1038/ncomms8122 (PMC4479000; doi:10.1038/ncomms8122)
Supplement: Supplementary Information — Supplementary Figures 1-8, Supplementary Tables 1-2 [file ncomms8122-s1.pdf]

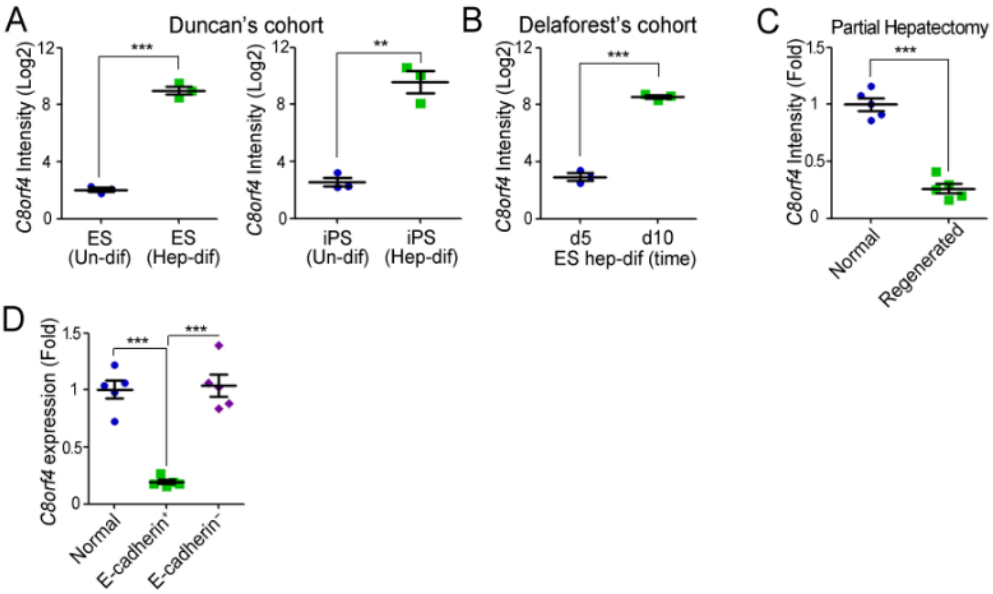

Supplementary Figure 1: *C8orf4* is lowly expressed in stem cells. (A) *C8orf4* expression levels were analyzed in undifferentiated ES cells and iPS cells provided by Duncan's cohort (GSE14897). Un-dif: undifferentiated; Hep-dif: hepatic-differentiation. (B) *C8orf4* expression levels were analyzed in day 5 and day 10 during hepatic-differentiation from ES cells (GSE25417). (C) Murine 2/3 partial hepatectomy (PH) model was established and expression levels of *C8orf4* was detected in normal livers and regenerated livers with stem cell features. (D) Murine liver progenitor cells derived from fetal liver were sorted for E-cadherin positive cells, then the progenitor cells with stem cell features and non-progenitor cells were examined for *C8orf4* expression. Student T test was used for statistical analysis, **, P < 0.01; ***, P < 0.001, data are shown as means ± SD.

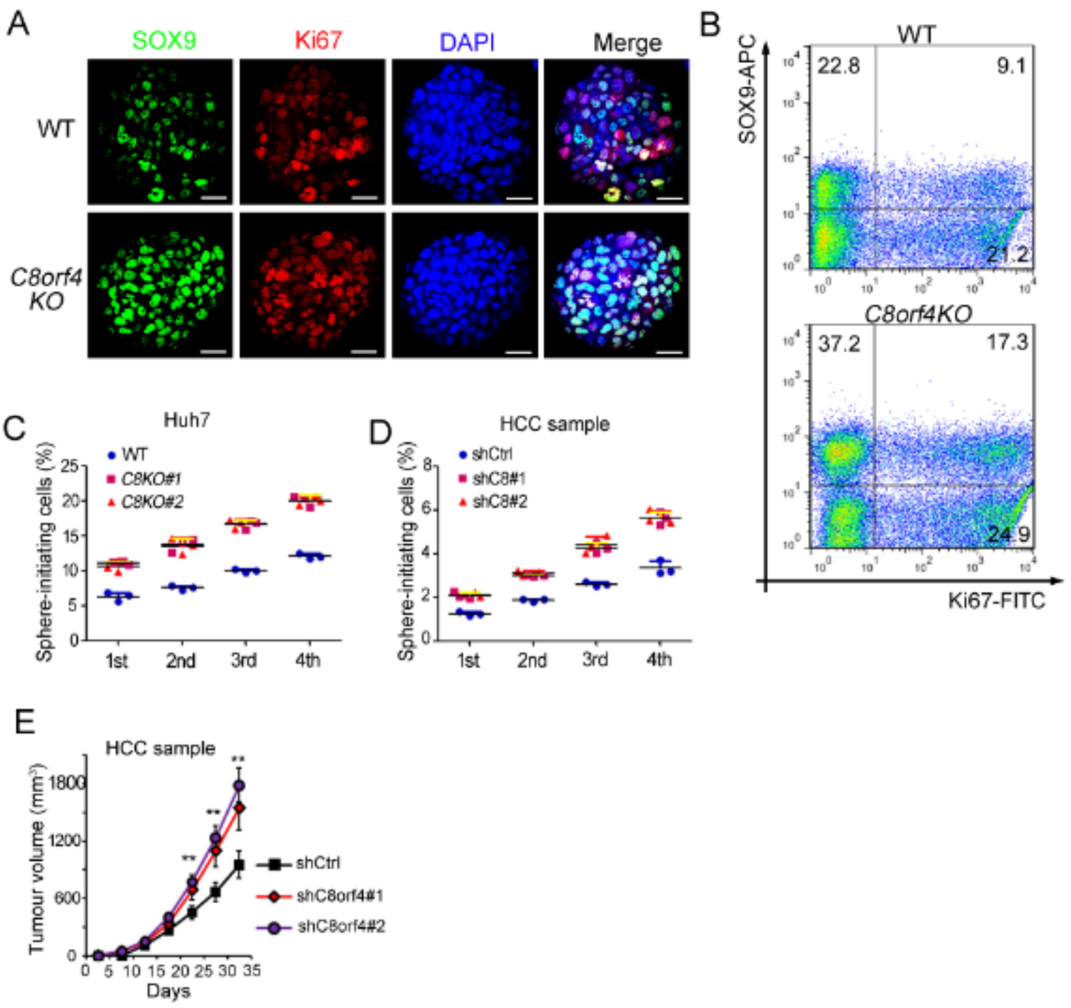

Supplementary Figure 2. C8orf4 inhibits self-renewal of liver CSCs. (A) *C8orf4* knockout and WT Huh7 sphere cells were stained with anti-SOX9 and anti-Ki67 antibodies, followed by confocal microscopy. *C8orf4 KO*: *C8orf4* knockout. Scale bars: 20um. (B) *C8orf4* knockout and WT Huh7 spheres were digested with Typsin/EDTA and then incubated with Ki67-FITC and SOX9-APC for immunofluorescence staining and analyzed by FACS analysis. (C, D) Serial sphere formation assays were performed using HCC cell line Huh7 cells (C) and HCC primary cells (D), and sphere formation ratios were shown. 1st: first generation; 2nd: second generation; 3rd: third generation; 4th: fourth generation; *C8KO*: *C8orf4* knockout. (E) 1×10^6 C8orf4 depleted and control primary cells were injected into BALB/c nude mice. Tumour sizes were measured every five days. Student T test was used for statistical analysis, *, $P < 0.05$; **, $P < 0.01$, data are shown as means ± SD.

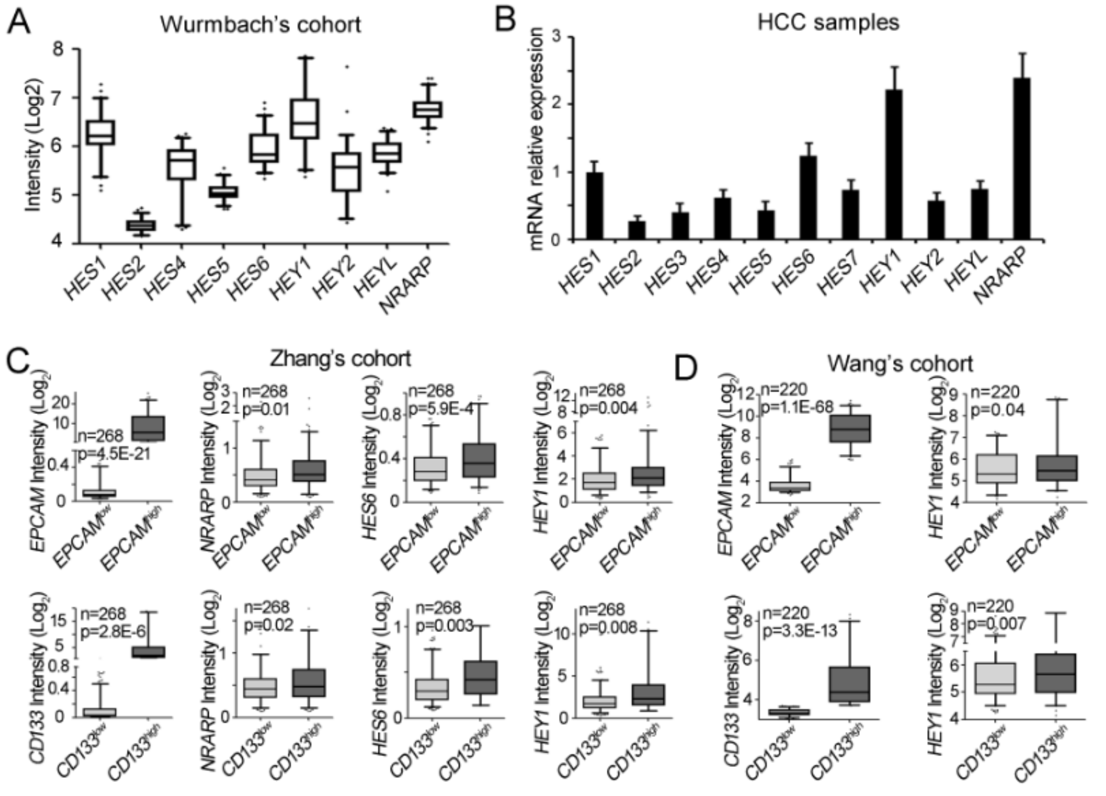

Supplementary Figure 3. C8orf4 inhibits NOTCH signaling. (A)Expression levels of NOTCH target genes (GSE6764) were analyzed by R language. Data are shown as box and whisker plot (5-95 percentile). (B) The expression levels of NOTCH target genes in 16 HCC samples were analyzed by realtime PCR. (C) *NRARP*, *HES6* and *HEY1* were highly expressed in *EPCAM*^{high} samples (upper panel) and *CD133*^{high} samples (lower panel) derived from Zhang's cohort. (D) *HEY1* expression was positively related to expression levels of *EPCAM* (upper penal) and *CD133* (lower penal) by analysis of Wang's cohort. For A, C, D, data are shown as box and whisker plot. Box: interquartile range; horizontal line within box: median; whiskers: 5-95 percentile. For B, data are shown as means ± standard deviation (SD).

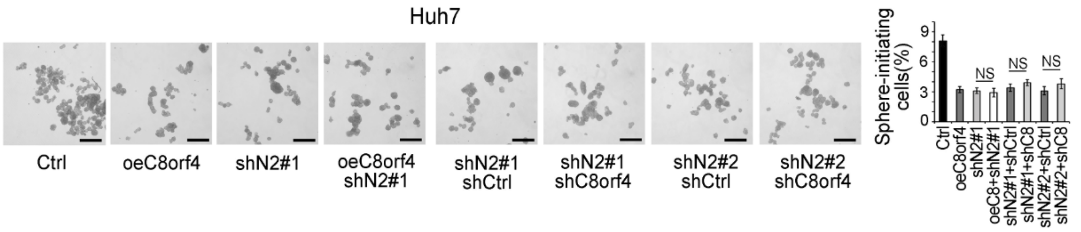

Supplementary Figure 4. C8orf4 inhibited self-renewal of CSC via NOTCH signaling. Sphere formation capacity was examined in differently treated Huh7 cells. Representative images are in the left panel and statistical results are shown as means ± SD (right panel). Data represent at least four independent experiments. Student T test was used for statistical analysis, NS: no significance (P>0.05).

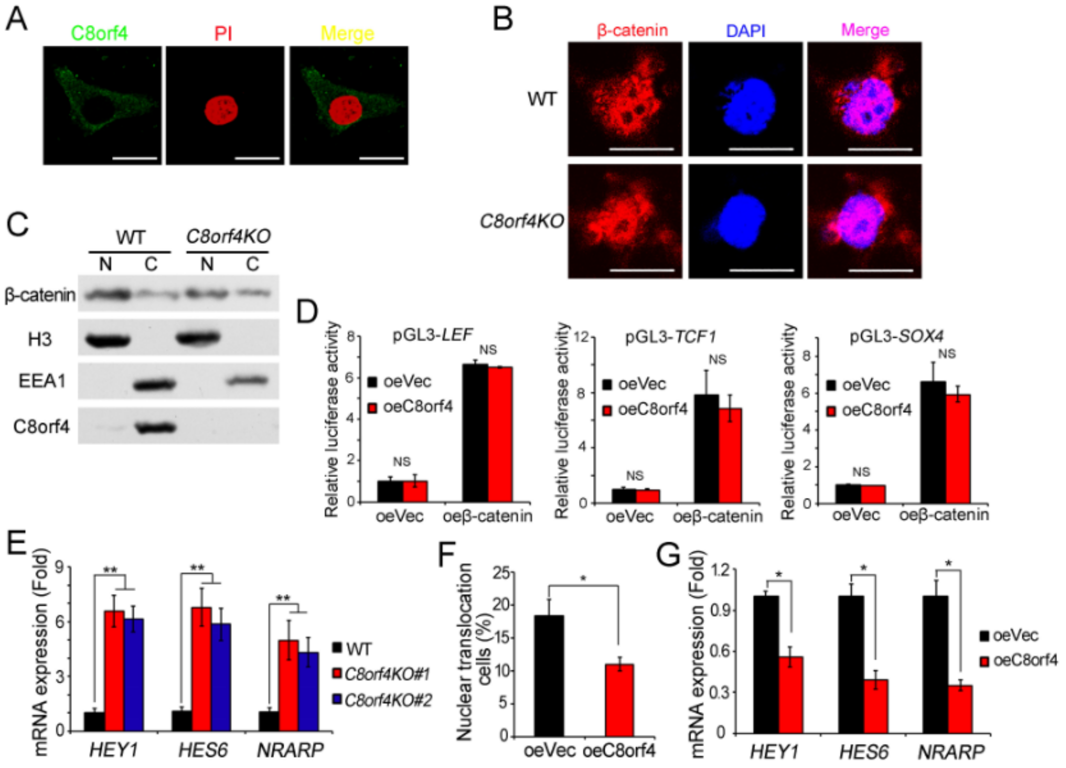

Supplementary Figure 5. *C8orf4* knockout causes nuclear translocation of N2ICD. (A) C8orf4 resides in the cytoplasm of Hep3B cells. Hep3B cells were permeabilized and stained with anti-C8orf4 antibody, then counterstained with PI for confocal microscopy. (B) *C8orf4* knockout and WT cells were stained with anti-β-catenin antibody for immunofluorescence staining. Scale bars: 10μm. (C) *C8orf4* knockout and WT cells were fractionated to nuclear and cytoplasmic parts followed by immunoblotting. (D) *LEF1*, *TCF1* and *SOX4* promoters were subcloned into pGL3 luciferase reporter plasmid, then co-transfected them into control (oeVec) and β-catenin overexpressing (oeβ-catenin) cells together with C8orf4 overexpression plasmid for 24 h, followed by luciferase activity assay. (E) *C8orf4* deficient and control tumours were obtained and examined expression levels of NOTCH2 target genes (*HEY1*, *HES6* and *NRARP*) by realtime PCR. *C8orf4KO*: *C8orf4* knockout. (F, G) C8orf4 overexpressing tumours were obtained, N2ICD nuclear translocation was analyzed using Immunohistochemical staining (F), and expression levels of NOTCH2 target genes were also examined by realtime PCR (G). oeC8orf4: C8orf4 overexpression. For A, B, scale bars: 10um. For D, E, F, G, student T test was used for statistical analysis, *, P < 0.05; **, P < 0.01, NS: no significance (P>0.05), data are shown as means ± SD.

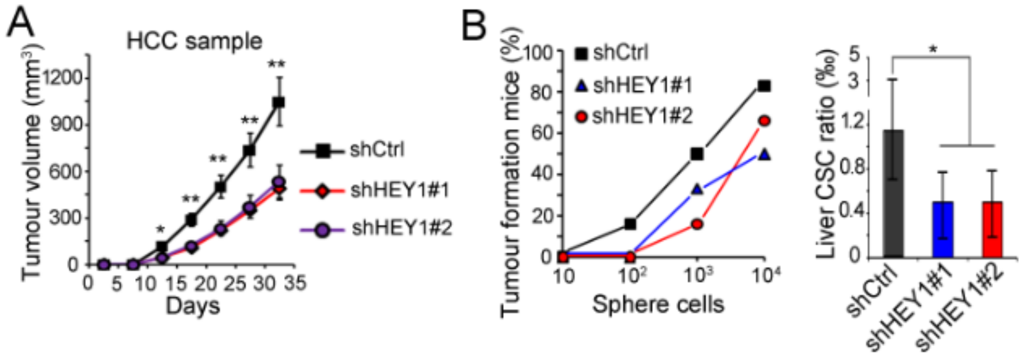

Supplementary Figure 6. HEY1 plays a critical role in tumour growth. (A) HEY1 depleted HCC primary cells were established using pSicoR lentivirus, then 1×10^6 HEY1 silenced and control cells were injected into BALB/c nude mice, and tumour volume was measured every five days. T test was used for statistical analysis, *, $P < 0.05$; **, $P < 0.01$, data are shown as means ± SD. (B) Oncospheres derived from HEY1 depleted and control cells were digested with trypsin/EDTA and 10, 10^2, 10^3 and 10^4 cells were injected into BALB/c mice, and tumour formation was observed at the third month. Percentages of tumour formation mice were calculated (left panel) and frequencies of tumour initiating cells were calculated using extreme limiting dilution analysis (right panel). Error bars represent the 95% confidence intervals of the estimation.

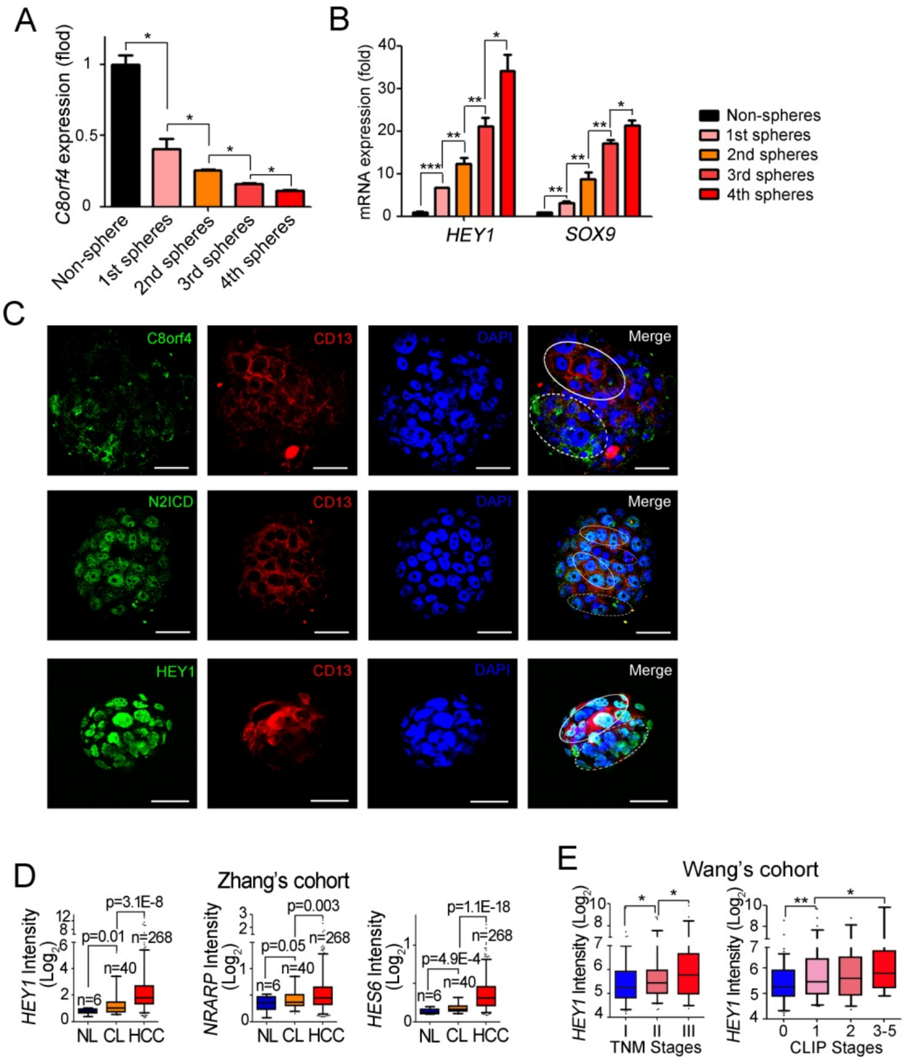

Supplementary Figure 7. C8orf4-N2ICDnucHEY1+ cells represent a subset of liver CSCs. (A, B) Expression levels of *C8orf4*, *HEY1* and *SOX9* in serial passage spheres. Serial sphere formation assays were performed using HCC primary cells, then spheres were collected and expression levels of *C8orf4* (A), *HEY1* and *SOX9* (B) were examined using realtime PCR. 1st: first generation; 2nd: second generation; 3rd: third generation; 4th: fourth generation. (C) The 4th generation HCC spheres were co-stained with CD13 and C8orf4 (upper), N2ICD (middle) and HEY1 (lower), followed by confocal microscopy. Solid line indicates liver CSC cells, and dotted line denotes non-CSC cells. (D) Expression levels of NOTCH target genes were elevated in HCC tumours derived from Zhang's cohort (GSE25097). (E) *HEY1* expression levels were consistent with clinical severity of HCC patients analyzed by Wang's cohort. TNM: tumour node metastasis; CLIP: cancer of the liver Italian program. For C, scale bars: 20 μm. For A, B, Student T test was used for statistical analysis, *, P< 0.05; **, P< 0.01; ***, P< 0.001, data are shown as means ± SD. For D, E, data are shown as box and whisker plot. Box: interquartile range; horizontal line within box: median; whiskers: 5-95 percentile.

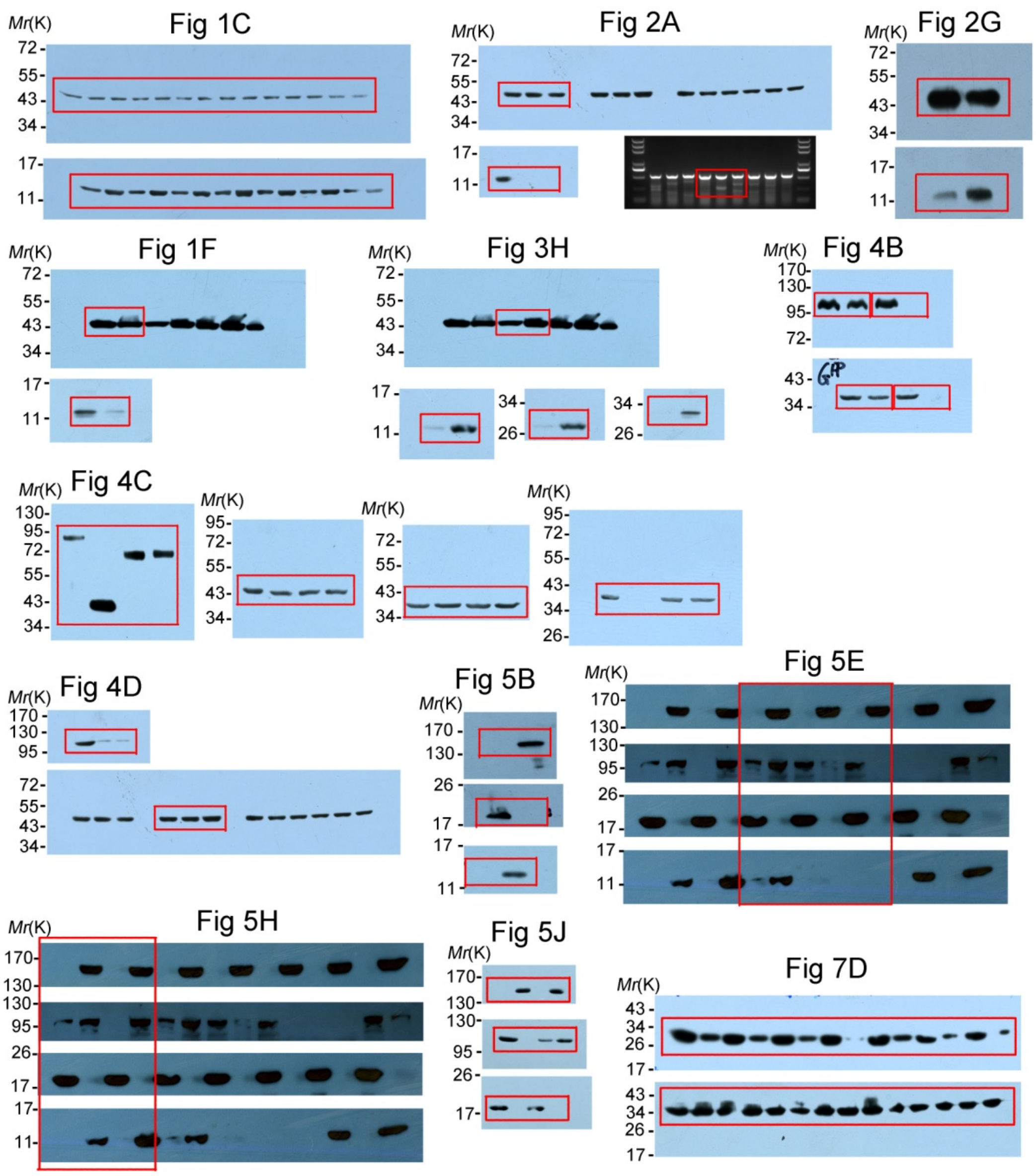

Supplementary Figure 8: Full blots of figures. The red sections indicate blot results shown in the indicated figures.

Supplementary Table 1. Realtime PCR primers used in this study.

Primers	Sequences
18S rRNA (Forward)	5’-AACCCGTTGAACCCCATT-3’
18S rRNA (Reverse)	5’-CCATCCAATCGGTAGTAGCG-3’
Actin (Forward)	5’-TCCATCATGAAGTGTGACGT-3’
Actin (Reverse)	5’-GAGCAATGATCTTGATCTTCAT-3’
C8orf4 (Forward)	5’-CAAGCCATCATCATGTCCAC-3’
C8orf4 (Reverse)	5’-GTTGCCCACGGCTTTCTTAC-3’
CD133 (Forward)	5’-AGTCGGAAACTGGCAGATAGC-3’
CD133 (Reverse)	5’-GGTAGTGTTGTACTGGGCCAAT-3’
GLI1 (Forward)	5’-TGGATATGATGGTTGGCAAGTG-3’
GLI1 (Reverse)	5’-ACAGACTCAGGCTCAGGCTTCT-3’
GLI3 (Forward)	5’-GAAGTGCTCCACTCGAACAGA-3’
GLI3 (Reverse)	5’-GTGGCTGCATAGTGATTGCG-3’
Patched (Forward)	5’-CCACAGAAGCGCTCCTACA-3’
Patched (Reverse)	5’-CTGTAATTTCGCCCCTTCC-3’
TCF1 (Forward)	5’-CACGGGCAAACACTACGGT-3’
TCF1 (Reverse)	5’-TTGACCTTCGAGTGCTGATCC-3’
CCND1 (Forward)	5’-GCTGCGAAGTGGAAACCATC-3’
CCND1 (Reverse)	5’-CCTCCTTCTGCACACATTTGAA-3’
MYC (Forward)	5’-GGCTCCTGGCAAAAGGTCA-3’
MYC (Reverse)	5’-CTGCGTAGTTGTGCTGATGT-3’
HES1 (Forward)	5’-TCAACACGACACCGGATAAAC-3’
HES1 (Reverse)	5’-GCCGCGAGCTATCTTTCTTCA-3’
HES6 (Forward)	5’-AGCAGGAGCCTGACTCAGTT-3’
HES6 (Reverse)	5’-AGCTCCTGAACCATCTGCTC-3’
HEY1 (Forward)	5’-GTTCGGCTCTAGGTTCCATGT-3’
HEY1 (Reverse)	5’-CGTCGGCGCTTCTCAATTATTC-3’
NRARP (Forward)	5’-CACGGGGTGATCACTGCTAA-3’
NRARP (Reverse)	5’-CGCTGGGCTACAGGTCAATA-3’
NOTCH1 (Forward)	5’-TTGGGAGGAGCAGATTTTTG-3’
NOTCH1 (Reverse)	5’-CACTGGCATGACACACAACA-3’
NOTCH2 (Forward)	5’-CAACTCGATGAGTGTGCGTC-3’
NOTCH2 (Reverse)	5’-ATGCCCTGGATGGAAAATGGA-3’
NOTCH3 (Forward)	5’-AGATTCTCATCCGAAACCGCTCTA-3’
NOTCH3 (Reverse)	5’-GGGGTCTCCTCCTTGCTATCCTG-3’
NOTCH4 (Forward)	5’-GCGGAGGCAGGGTCTCAACGGATG-3’
NOTCH4 (Reverse)	5’-AGGAGGCGGGATCGGAATGT-3’
CD13 (Forward)	5’-GACCAAAGTAAAGCGTGGAATCG-3’
CD13 (Reverse)	5’-TCTCAGCGTCACCCGGTAG-3’

Supplementary Table 2. shRNA sequence used in this study.

shRNA	Sequences
**shC8orf4#1**	5'-GCACCAAGCCATCATCATG-3'
**shC8orf4#2**	5'-GACAGACCAAGAATCACTA-3'
**shNOTCH2#1**	5'-GGATAAGATTGGAGGCTTC-3'
**shNOTCH2#2**	5'-GGAGAACATTGACAACTGT-3'
**shHEY1#1**	5'-GCATCATTGAAGGACTAGA-3'
**shHEY1#2**	5'-GACGGAGAGGAATAATTGA-3'
**shNRARP#1**	5'-GCTCTATCTCATCACCAAG-3'
**shNRARP#2**	5'-CTGCAGAACATGACCAAC-3'
